# Drug metabolism and clearance system in tumor cells of patients with multiple myeloma

**DOI:** 10.18632/oncotarget.3237

**Published:** 2014-12-26

**Authors:** Wafa Hassen, Alboukadel Kassambara, Thierry Reme, Surinder Sahota, Anja Seckinger, Laure Vincent, Guillaume Cartron, Jérôme Moreaux, Dirk Hose, Bernard Klein

**Affiliations:** ^1^ Institute of Human Genetics, CNRS-UPR1142, Montpellier, France; ^2^ High Institute of Biotechnology of Monastir, University of Monastir, Tunisia; ^3^ CHU Montpellier, Laboratory for Monitoring Innovative Therapies, Department of Biological Haematology, Montpellier, F-34295 France; ^4^ Cancer Sciences Unit, Faculty of Medicine, University of Southampton, SO17 1BJ, UK; ^5^ Medizinische Klinik V, Universitätsklinikum Heidelberg, Heidelberg, Germany; ^6^ Nationales Centrum für Tumorerkrankungen, Heidelberg, Germany; ^7^ CHU Montpellier, Department of Clinical Hematology, Montpellier, F-34295, France; ^8^ University of Montpellier 1, UFR Medicine, Montpellier, F-34967, France

**Keywords:** Multiple Myeloma, Drug Metabolism and Clearance, Prognosis

## Abstract

Resistance to chemotherapy is a major limitation of cancer treatments with several molecular mechanisms involved, in particular altered local drug metabolism and detoxification process. The role of drug metabolism and clearance system has not been satisfactorily investigated in Multiple Myeloma (MM), a malignant plasma cell cancer for which a majority of patients escapes treatment. The expression of 350 genes encoding for uptake carriers, xenobiotic receptors, phase I and II Drug Metabolizing Enzymes (DMEs) and efflux transporters was interrogated in MM cells (MMCs) of newly-diagnosed patients in relation to their event free survival. MMCs of patients with a favourable outcome have an increased expression of genes coding for xenobiotic receptors (*RXR*α, *LXR*, *CAR* and *FXR*) and accordingly of their gene targets, influx transporters and phase I/II DMEs. On the contrary, MMCs of patients with unfavourable outcome displayed a global down regulation of genes coding for xenobiotic receptors and the downstream detoxification genes but had a high expression of genes coding for *ARNT* and *Nrf2* pathways and ABC transporters. Altogether, these data suggests *ARNT* and *Nrf2* pathways could be involved in MM primary resistance and that targeting *RXRα, PXR, LXR* and *FXR* through agonists could open new perspectives to alleviate or reverse MM drug resistance.

## INTRODUCTION

Multiple Myeloma (MM) is a malignant plasma cell disease accounting for approximately 10% of haematological malignancies, with 25,000 new patients per year in the EU and a median survival of five years [1]. The disease develops primarily in the bone marrow and is associated with end organ damages including bone lesions, renal failure and anaemia [2]. Although significant advances have been made, the current treatment regimens do not cure the majority of patients who repeatedly relapse until they succumb to the disease [3]. Resistance to chemotherapy is a major hurdle limiting the efficacy of MM treatment. Anticancer drugs resistance can be innate –primary- or acquired over time following exposure to the drug and involves diverse molecular mechanisms, in particular, altered local drug metabolism and detoxification process is a major barrier that lies between chemotherapeutic agents and their intended curative potential [4,5].

Drug metabolism and clearance (DMC) system is a defense system that imports, sensors, inactivates and excretes chemicals (xenobiotics) from a variety of sources including dietary components, environmental pollutants and drugs that can profoundly impair the structure and function of cells and tissues. After drug uptake, DMC system proceeds through enzymatic conversion of xenobiotics into more water-soluble metabolites that are better effluxed from the cell through membrane transporters and discharged into urinary and biliary systems [6,7].

Drug sensing is mediated by members of the superfamily of nuclear receptors including Pregnane X Receptor (*PXR, NR1I2*), Constitutive Androstane Receptor (*CAR, NR1I3*), Liver X Receptor (*LXR, NR1H3*) and the Farnesol X Receptor (*FXR, NR1H4*) as well as some cytosolic ligand-activated transcription factors, *i.e.,* Hepatocyte Nuclear factor 4 (*HNF4*), Nuclear factor-erythroid 2p45-related factor 2 (*Nrf2*), Hypoxia inducing factors (*HIF1α, HIF3 α*), Metal transcription factors (*MTF1, MTF2*) and the Aryl hydrocarbon Receptor (*AhR*). Theses xenobiotic receptors coordinately regulate the defense against nearly all xenochemicals and often share common properties particularly broad ligand specificity and diverse often-overlapping spectra of target genes [6,8].

After xenobiotic binding, these receptors translocate to the nucleus and govern the tandem expression of genes encoding for phase I and II Drug Metabolizing Enzymes (DMEs) and transporters. Phase I DMEs consist primarily of oxidases, reductases and dehydrogenases that detoxify xenobiotics by introducing, modifying or unmasking a polar functional group into xenobiotics, respectively. Cytochrome P450s (*CYP450*s) are the main Phase I DMEs detoxifying a vast number of xenobiotics, including 80% of drugs used in clinic [9,10]. Phase II DMEs subsequently conjugate highly polar endogenous ligands (glutathione, sulfate, glucoronide, amino-acid, methyl and acetyl) to phase I metabolites of xenobiotics, giving rise to more hydrophilic compounds, which can be excreted out of the cell. Glutathione S-transferases (*GST*), UDP-glucoronosyltransferase (*UGT*) and sulfotransferases (*SULT*) constitute the major routes of conjugation [11,12]. Both parental xenobiotics and their metabolites can finally be exported out of the cell through xenobiotic transporters (Phase III), which mediate translocation of chemicals into and out of cells. Drug transporters constitute a superfamily of specialized proteins that span cell membrane bilayers and mediate translocation of chemicals into and out of cells. Depending on the source of energy, these transporters belong either to the family of ATP binding cassette (ABC) transporters that utilizes ATP hydrolysis-generated energy or to the subfamilies of Solute Carriers (SLC) driven by an exchange or cotransport of intracellular and/or extracellular ions with the substrate. ABC transporters comprise seven families with about 20 carriers involved in drug transport and mediate only unidirectional efflux. The SLC comprise 52 families with many drug carriers involved in both drug uptake (Phase 0) and efflux. The role of these xenobiotic transporters is crucial, dictating the circulating and cellular levels of drugs and subsequently their cytotoxic/therapeutic effects [13,14].

Unsurprisingly, DMC system has been demonstrated to compromise the efficacy of cancer chemotherapy and lead to treatment failure through promoting the metabolism and the elimination of chemotherapeutic agents. Much attention has been directed toward the role of ABC transporters since numerous studies have established a causative link between high expression of ABC proteins and worse clinical outcome and refractory disease [15]. Similarly, the expression of phase I / II DMEs have also been proved to modulate chemotherapeutic efficacy [5]. More recently, several studies have thus shown that the activation of *PXR, AhR, Nrf2, HIF1α or PPARs* play a critical role in altering the therapeutic response through reducing active drug concentration within tumor cells [4,16]. Meanwhile, emerging evidences suggest that the activation of DMC system in response to cancer drugs could also enhance chemosensitivity. As such, the drug biotransformation through phase I DMEs does not always yield pharmacologically inactive metabolites and could instead produce highly active toxic metabolites in a common process referred to as bioactivation [17]. Moreover, xenobiotic receptors functions are tissue/context-specific manner and their activation in different cancer settings have been reported to be pro-apoptotic, anti-proliferative and antitumoral [18].

Despite the relevance of DMC process for the effectiveness or failure of chemotherapy, its contribution to MM pathology and prognosis have been poorly investigated with much concern given to the study of single nucleotide polymorphism (SNP) of phase I and II enzymes, transporters and some xenobiotic receptors genes [19]. To this end, we have looked for the expression of 350 genes encoding for uptake carriers, xenobiotic receptors, phase I/II DMEs and efflux transporters in MM cells (MMCs) of patients with newly diagnosed MM, in relation with their clinical outcome (relapse and survival). This study shows that MMCs of patients with a better survival are metabolically competent and display an increased expression of genes coding for several xenobiotic receptors and their downstream target genes among influx and efflux transporters and phase I/II DMEs. On the other hand, MMCs of patients with poor outcome exhibit global down regulation of DMC genes but overexpressed genes coding for *Nrf2* and *ARNT* pathways and several members of ABC transporter family suggesting that *Nrf2* and *ARNT* pathways are likely to be key players of MM primary resistance.

## RESULTS

### 40 Drug Metabolism and Clearance genes are prognostic to EFS in MM patients

The expression of 40 genes among a consensus list of 350 genes coding for DMC system (Supplementary Table S1) was found to be prognostic for Event Free Survival (EFS) in patients of the HM cohort using a Cox analysis. Fourteen genes were associated with a good prognosis and 26 with a bad one. The 14 good prognostic genes encode for 4 xenobiotic receptors (*RXRα, HNF1α, MTF1* and *FXR*), 4 phase I DMEs (*CYP46A1, CYP1B1, NQO2, XHD*), 2 phase II DMEs (*SAT1, BAAT*) and 4 SLC members (*SLC2A1, SLC2A3, SLC22A4, SLC22A15*) (Table 1). The 26 bad prognostic genes encode for 12 transporters (including 7 members of ABC family - *ABCB1, ABCB2, ABCB10, ABCD1, ABCD2, ABCE1, ABCF3* - and 4 members of SLC family - *SLC38A5, SLC16A1, SLCO5A1 and SLC19A2)*, the Aryl hydrocarbon Receptor Nuclear Translocator (*ARNT*), *MTF2* and *Keap1-* the cytosolic regulator of *Nrf2* (Table 2).

**Table 1:** Good prognostic genes for patients of the HM cohort The value of the expression of each of the 350 DMC genes for predicting the EFS of the newly-diagnosed patients of the HM cohort was looked for using a Cox univariate analysis. Data are the beta coefficients, the hazard ratios and P values of the Cox model. Genes are ranked according to increasing P values.

Probe set	Name	Beta Coefficient	HR	P value
202449_s_at	*RXRA*	−0.36	0.7	0.0011
210515_at	*HNF1A*	−0.29	0.75	0.0082
220331_at	*CYP46A1*	−0.28	0.75	0.0086
203455_s_at	*SAT1*	−0.26	0.77	0.013
201250_s_at	*SLC2A1*	−0.25	0.78	0.016
202436_s_at	*CYP1B1*	−0.3	0.74	0.024
206913_at	*BAAT*	−0.23	0.79	0.025
202499_s_at	*SLC2A3*	−0.33	0.72	0.028
228497_at	*SLC22A15*	−0.24	0.79	0.033
205322_s_at	*MTF1*	−0.23	0.79	0.034
206340_at	*NR1H4*	−0.22	0.81	0.037
203814_s_at	*NQO2*	−0.24	0.79	0.038
205896_at	*SLC22A4*	−0.27	0.76	0.042
210301_at	*XDH*	−0.19	0.82	0.049

**Table 2:** Bad prognostic genes for patients of the HM cohort The value of the expression of each of the 350 DMC genes for predicting the EFS of the newly-diagnosed patients of the HM cohort was looked for using a Cox univariate analysis. Data are the beta coefficient, the hazard ratio and P value of the Cox model. Genes are ranked according to increasing P values.

Probe set	Name	Beta Coefficient	HR	p-Value
203302_at	*DCK*	0.32	1.4	1e-04
202854_at	*HPRT1*	0.35	1.4	6e-04
223320_s_at	*ABCB10*	0.29	1.3	0.00074
230619_at	*ARNT*	0.25	1.3	0.00096
219565_at	*CYP20A1*	0.30	1.4	0.0014
209646_x_at	*ALDH1B1*	0.3	1.3	0.0016
224918_x_at	*MGST1*	0.26	1.3	0.0021
234973_at	*SLC38A5*	0.25	1.3	0.0022
205073_at	*CYP2J2*	0.28	1.3	0.0026
206756_at	*CHST7*	0.25	1.3	0.0028
202307_s_at	*TAP1*	0.27	1.3	0.0039
202236_s_at	*SLC16A1*	0.25	1.3	0.0066
201612_at	*ALDH9A1*	0.24	1.3	0.0074
220984_s_at	*SLCO5A1*	0.25	1.3	0.0081
201872_s_at	*ABCE1*	0.25	1.3	0.0087
202180_s_at	*MVP*	0.23	1.3	0.012
202850_at	*ABCD3*	0.24	1.3	0.013
207583_at	*ABCD2*	0.24	1.3	0.016
202417_at	*KEAP1*	0.25	1.3	0.016
202589_at	*TYMS*	0.19	1.2	0.017
236597_at	*UGT3A1*	0.21	1.2	0.022
209681_at	*SLC19A2*	0.2	1.2	0.026
202394_s_at	*ABCF3*	0.22	1.2	0.029
202275_at	*G6PD*	0.20	1.2	0.03
209993_at	*ABCB1*	0.21	1.2	0.033
203345_s_at	*MTF2*	0.2	1.2	0.049

### The Drug Metabolism and Clearance score splits patients of two independent cohorts into three groups with different EFS and OS

**Figure 1:**
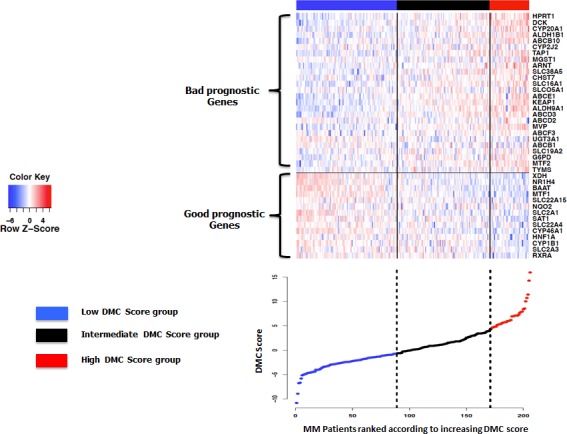
Heatmap of supervised clustering of the 40 prognostic genes for EFS along the 206 patients of the HM cohort ranked according increasing DMC score A k-means function was used to identify the −0.673 and 4.24 cutoff points to split patients into 3 groups with a low, intermediate and high DMC score.

The prognostic information of these 40 DMC genes was summed within a single parameter - a DMC score - as indicated in the Methods section. DMC score ranged from −10.79 to 15.97 in the 206 MMCs of the patients of the HM cohort and the higher the DMC score is, the worse the outcome is. Running an unsupervised clustering of the 40 prognostic DMC genes along the 206 patients ranked according to increasing DMC score, genes were split into 2 clusters: a cluster comprising bad prognostic genes mainly overexpressed in MMCs with the highest DMC scores (bad prognosis) and a second cluster with the good prognostic ones overexpressed in MMCs with the lowest DMC scores (good prognosis). MMCs with intermediate DMC scores variably expressed both bad and good prognosis DMC genes (Figure 1). To delineate these 3 patient groups, a k-means clustering (3 groups, 200 runs) was used splitting patients into a low DMC score group comprising 43% of the patients (−10.79 ≤ DMC score < −0.673), an intermediate DMC score group (40% of the patients, −0.673 ≤ DMC score < 4.24) and a high DMC score group (17% of the patients, 4.24 ≤ DMC score < 15.97) (Figure 1).

**Figure 2:**
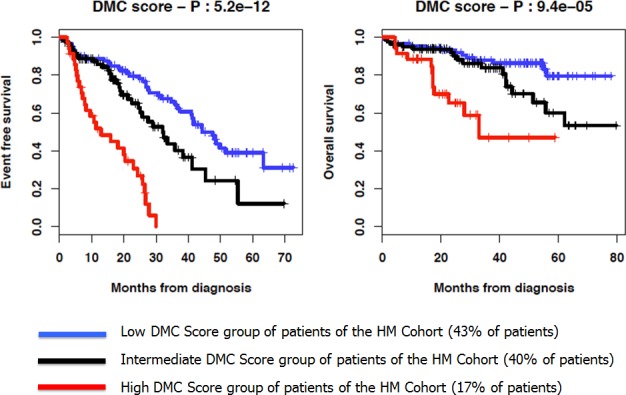
Kaplan-Meier curves of the EFS and OS of the 3 DMC score groups of patients of the HM cohort

As illustrated in Figure 2, patients of the HM cohort from the 3 DMC groups had different EFS and Overall Survival (OS). The median EFS were 12.9, 32 and 47.6 months for the high, intermediate and low DMC score groups, respectively (P = 5.2 × 10^−12^) (Figure 2). The median OS was 32.9 months for the high DMC score group and not reached for both intermediate and low DMC score groups (P = 9.4 × 10^−5^, Figure 2).

**Figure 3:**
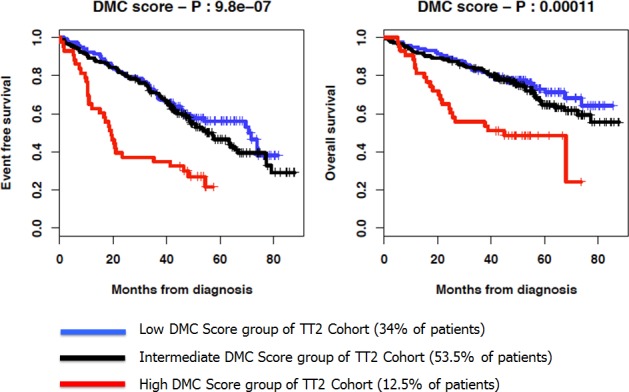
Kaplan-Meier curves of the EFS and OS of the 3 DMC score groups of patients of the UAMS-TT2 cohort

Using the cutoff points designed with the HM cohort (− 0.673 and 4.24), patients of the independent UAMS-TT2 cohort were similarly split into high, intermediate and low DMC score groups comprising 12.5, 34% and 53.5% of patients, respectively. The median EFS of patients of the high DMC score group was 19.3 months, 3- and 3.5-fold shorter than that of the low and intermediate DMC score patients, respectively (P = 9.8 × 10^−7^) (Figure 3). The median EFS of the intermediate and low DMC score groups were not significantly different. Furthermore, the median OS of UAMS-TT2 patients of the high DMC score group was 45 months and not-reached for the low and intermediate DMC score groups (P = 1.1 × 10^−4^) (Figure 3).

### Drug Metabolism and Clearance profile in MMCs of patients with bad versus good prognosis

The above analysis shows that the 40 DMC prognostic genes could split patients of 2 independent cohorts into at least two groups with a different EFS and OS. We looked for a differential expression of all 350 DMC genes between these groups. The intermediate DMC group was not considered because MMCs of these patients variably expressed both good and bad prognosis DMC genes. Using a SAM supervised analysis (2 fold change, FDR ≤ 0.05), 101 of the 350 DMC genes had an increased expression in low DMC score MMCs (good prognosis group) and only 14 in high DMC score MMCs (bad prognosis group) (Tables 3 and 4). The 101 genes whose expression is increased in the low DMC score group include genes coding for 7 xenobiotic receptors, particularly *FXR* (4.5-fold increase), *HIF3α* (4.1-fold increase), *HNF4 α* (4-fold increase), *CAR* (3.1-fold increase), *MTF1* (2.7-fold increase). These 101 low DMC score group genes include 30 of the known 47 CYP genes including members of the CYP2 (*CYP2B6, CYP2C9, CYP2E1* and *CYP2D6*) and CYP3 families (*CYP3A4, CYP3A5* and *CYP3A7*), 19 genes coding for SLC members and 11 genes for ABC transporters (Table 3). The 14 genes whose expression is up-regulated in the high DMC score group comprise genes coding for the *ARNT* transcription factor, Keap1 the co-regulator of Nrf2, 3 ABC members (*ABCD3, ABCE1, ABCB2/TAP1*), 1 SLC member (*SLC16A1*) and 1 CYP450 (*CYP20A1*) (Table 4).

**Table 3:** Genes up regulated in low DMC Score group The expression of the 350 DMC genes in MMCs of patients of the two low versus high DMC score groups (HM cohort, −10.79. DMC score < −0.673 and 4.24 ≤ DMC score ≤ 15.97) was compared using a SAM supervised analysis (2 fold change, FDR ≤0.05). Data are the list of the 101 genes whose expression in increased in MMCs of patients with low DMC score and their fold change in expression between low and high score MMCs.

Probe set	Name	Fold Change
207225_at	*AANAT*	2.24
210082_at	*ABCA4*	2.01
217504_at	*ABCA6*	2.23
219577_s_at	*ABCA7*	2.17
242541_at	*ABCA9*	4.1
1569072_s_at	*ABCB5*	4.87
1554911_at	*ABCC11*	3.82
1553410_a_at	*ABCC12*	2.29
239217_x_at	*ABCC3*	3.89
210245_at	*ABCC8*	2.47
208462_s_at	*ABCC9*	2.75
207593_at	*ABCG4*	2.41
234197_at	*ACSM1*	2.91
207820_at	*ADH1A*	2.22
223781_x_at	*ADH4*	2.87
210505_at	*ADH7*	2.96
227113_at	*ADHFE1*	2.88
210962_s_at	*AKAP9*	2.16
240435_at	*ALDH1A2*	2.75
211004_s_at	*ALDH3B1*	2.1
204942_s_at	*ALDH3B2*	2.27
205082_s_at	*AOX1*	2.33
206955_at	*AQP7*	2.23
223652_at	*AS3MT*	2.8
206913_at	*BAAT*	3.86
205627_at	*CDA*	2.08
220446_s_at	*CHST4*	2.63
221164_x_at	*CHST5*	2.58
224400_s_at	*CHST9*	4.34
205502_at	*CYP17A1*	2.48
203475_at	*CYP19A1*	2.36
205749_at	*CYP1A1*	3.06
202436_s_at	*CYP1B1*	2.68
206504_at	*CYP24A1*	2.5
208327_at	*CYP2A13*	2.04
211295_x_at	*CYP2A6*	2.26
207718_x_at	*CYP2A7*	2.15
206755_at	*CYP2B6*	4.08
210272_at	*CYP2B7P1*	2.28
208126_s_at	*CYP2C18*	2.09
216058_s_at	*CYP2C19*	2.13
216025_x_at	*CYP2C9*	3.75
217468_at	*CYP2D6*	2.3
209975_at	*CYP2E1*	3.5
220562_at	*CYP2W1*	2.67
244407_at	*CYP39A1*	2.43
205998_x_at	*CYP3A4*	2.11
211440_x_at	*CYP3A43*	2.24
214234_s_at	*CYP3A5*	2.61
205939_at	*CYP3A7*	2.29
220331_at	*CYP46A1*	3.21
211231_x_at	*CYP4A11*	2.27
1555497_a_at	*CYP4B1*	2.23
206153_at	*CYP4F11*	2.78
206539_s_at	*CYP4F12*	2.87
210452_x_at	*CYP4F2*	3.24
237395_at	*CYP4Z1*	3.2
207386_at	*CYP7B1*	3.22
232494_at	*CYP8B1*	2.21
228268_at	*FMO2*	3.55
206930_at	*GLYAT*	5.33
205752_s_at	*GSTM5*	2.74
222124_at	*HIF3A*	4.14
208429_x_at	*HNF4A*	4
204041_at	*MAOB*	4.38
205813_s_at	*MAT1A*	3.02
244122_at	*MGST3*	2.85
205322_s_at	*MTF1*	2.7
206797_at	*NAT2*	2.18
202237_at	*NNMT*	3.12
206410_at	*NR0B2*	2.59
206340_at	*NR1H4*	4.55
207007_at	*NR1I3*	3.11
206345_s_at	*PON1*	2.05
210367_s_at	*PTGES*	2.89
208131_s_at	*PTGIS*	2.17
205128_x_at	*PTGS1*	2.32
204748_at	*PTGS2*	3.46
217020_at	*RARB*	2.1
207185_at	*SLC10A1*	3.95
207095_at	*SLC10A2*	2.78
240159_at	*SLC15A2*	2.13
1552761_at	*SLC16A11*	2
204462_s_at	*SLC16A2*	2.59
220455_at	*SLC16A8*	4.77
237799_at	*SLC22A12*	2.1
207444_at	*SLC22A13*	2.64
232232_s_at	*SLC22A16*	2
220554_at	*SLC22A7*	3.52
231352_at	*SLC22A8*	3.6
207560_at	*SLC28A1*	3.06
216432_at	*SLC28A2*	2.3
220475_at	*SLC28A3*	3.15
1560149_at	*SLC29A2*	3.28
242773_at	*SLC5A1*	2.05
216603_at	*SLC7A8*	3.03
220135_s_at	*SLC7A9*	4.32
204368_at	*SLCO2A1*	4.13
207601_at	*SULT1B1*	2.01
219934_s_at	*SULT1E1*	2.61
210301_at	*XDH*	3.31

**Table 4:** Genes up regulated in High DMC Score group The expression of the 350 DMC genes in MMCs of patients of the high versus low DMC score groups (HM cohort, −10.79 ≤ DMC score < −0.673 and 4.24 ≤ DMC score ≤ 15.97) was compared using a SAM supervised analysis (2 fold change, FDR ≤ 0.05). Data are the list of the 14 genes whose expression in increased in MMCs of patients with high DMC score and their fold change in expression between high and low score MMCs.

Probe set	Name	Fold Change
202850_at	*ABCD3*	2.045
201872_s_at	*ABCE1*	2.115
209646_x_at	*ALDH1B1*	2.539
230619_at	*ARNT*	2.017
202024_at	*ASNA1*	2.059
219565_at	*CYP20A1*	2.027
203302_at	*DCK*	3.461
202275_at	*G6PD*	2.832
202854_at	*HPRT1*	2.114
202417_at	*KEAP1*	2.196
202180_s_at	*MVP*	2.28
202236_s_at	*SLC16A1*	2.566
202307_s_at	*TAP1*	2.317
209605_at	*TST*	2.025

**Figure 4:**
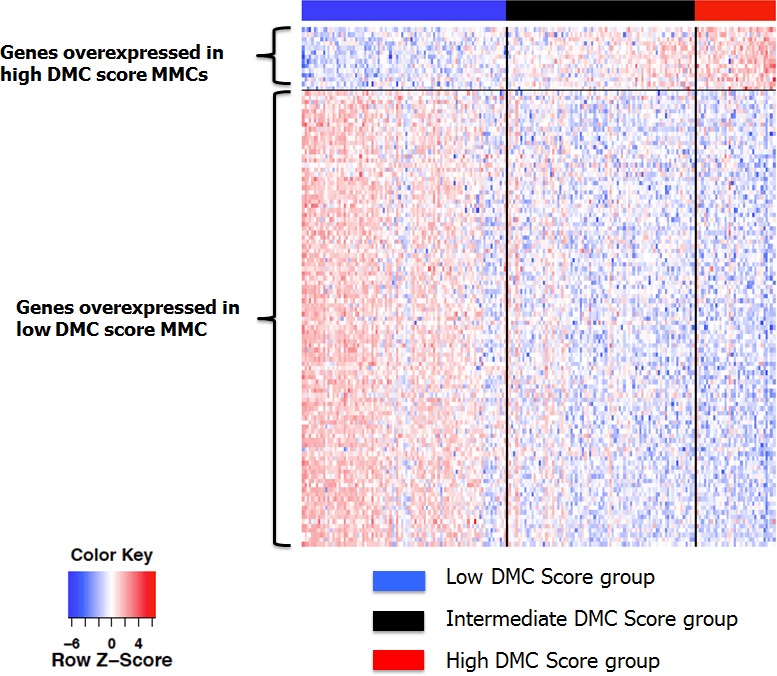
Heatmap of the supervised clustering of genes differentially expressed between low and high DMC score MMCs of patients of the HM cohort Patients are ranked according to increasing DMC score.

**Figure 5:**
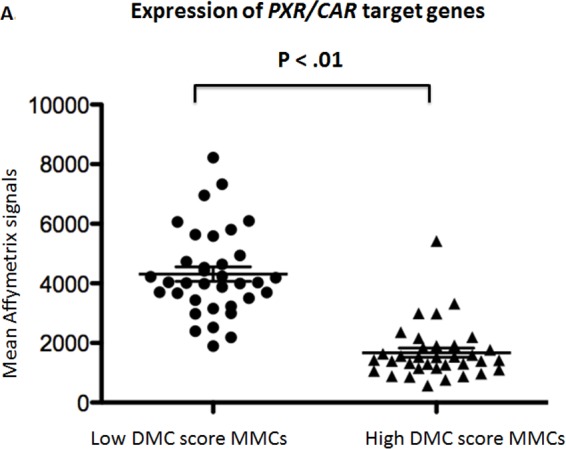

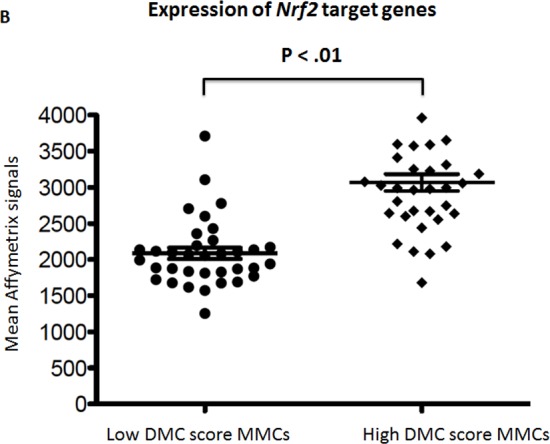
Expression of the target genes driven by *PXR/CAR* and *Nrf2* in low and high DMC score MMCs Data are the mean Affymetrix signals interrogating the target genes driven by PXR/CAR activation (A) or by Nrf2 (B) activation in MMCs of the patients of the low or high DMC score groups designed in Figure 1. The horizontal bars indicate the mean values ± SD of the expression of all target genes in each MMC group and these mean values were compared using a student t-test.

As illustrated by the supervised clustering of the expression of these 115 genes in MMCs of patients of the HM cohort ranked according to increasing DMC score (Figure 4), this data emphasizes that MMCs of the low DMC score group have higher abilities for biotransformation and detoxification of xenobiotics including drugs with regard to the high expression of a majority of xenobiotic receptors and of their downstream target genes (30% of DMC genes overexpressed) compared to MMCs of the high DMC score group (only 5% of DMC genes overexpressed). This is further evidenced by an Ingenuity Pathway Analysis which reveals an enrichment of genes coding for *PXR/CAR* pathways in the low DMC group and for *Nrf2* pathway in the high DMC group (data not shown). In addition, the expression of *PXR/CAR* target genes was higher in low DMC score MMCs compared to high DMC score ones and the reverse for *Nrf2* target genes (Figures 5A and 5B).

### Whole genome molecular portrait of MMCs of patients with bad versus good prognosis

**Figure 6:**
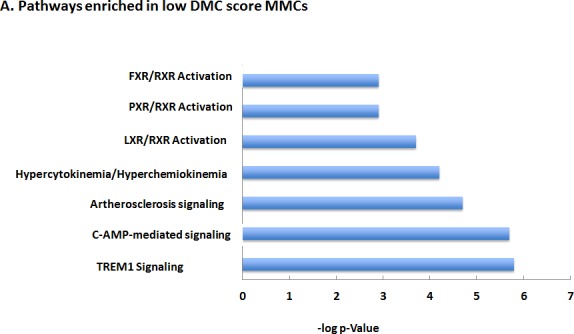

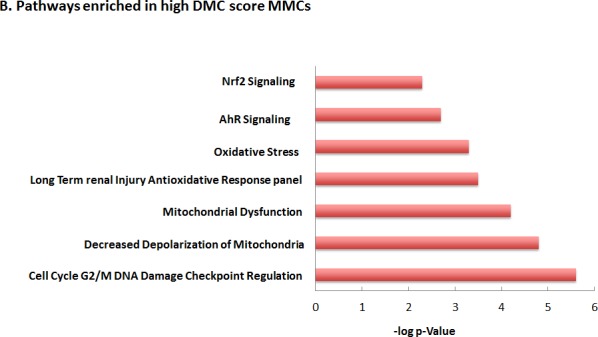
Major Pathways enriched in low (A) or high (B) DMC score MMCs The Ingenuity Pathway Analysis was used to identify the pathways encoded by the whole genome genes differentially expressed between low and high DMC score MMCs.

In order to get a better insight of the biological pathways delineating MMCs with low and a high DMC score, we looked for the differential expression of 12684 genes (variance ≥ 100) using a SAM supervised analysis (fold change ≥ 2, FDR ≤ 0.05, 1000 permutations) in MMCs of patients with low versus high DMC score (good versus bad prognosis). 2026 genes had their expression up regulated in low DMC score MMCs and 544 in high DMC score ones. Using Ingenuity Pathway Analysis, low DMC score MMCs showed an enrichment for pathways of the DMC system including *LXR, PXR, FXR*, Cytochrome P450 pathways, together with an enrichment for cytokine pathways (hyper cytokinemia, hyper chemiohinema and atherosclerosis signaling pathways) with *TREM1, STAT3, Rel A, CREB* and *ILβ* as upstream regulators (Figure 6A). High DMC score MMCs displayed prominently an enrichment for DNA Replication, Damage and Repair, Mitochondria dysfunction and oxidative stress response pathways (Figure 6B). Using the C2 Kegg collection (c2.cp.kegg.v.4.0) of the Gene Set Enrichment Analysis (GSEA) software, two modules of gene sets were enriched in high DMC score MMCs, the first one includes oxidative stress, mitochondrial dysfunction, unfolded protein response and proteasome deregulation with ubiquitin mediated proteolysis (Parkinson's disease, Oxidative Phosphorylation, Alzheimer disease, proteasome, Huntington disease) and the second module includes DNA repair, mismatch repair and base excision repair gene sets. The low DMC score MMCs showed enrichment for drug and xenobiotic metabolism, cytokine-cytokine receptor interaction, retinol and lipid metabolism and calcium signaling pathways.

## DISCUSSION

The current study shows that MMCs of patients with good clinical outcome, harvested prior to any treatment, overexpressed genes coding for several xenobiotic receptors (LXR, *CAR, FXR* and *RXRA*), and accordingly their downstream target genes coding for DMC actors (phase I/II DMEs, uptake and efflux transporters). On the contrary, MMCs of patients with bad outcome bear global down regulation of DMC system but highly expressed genes coding for the *ARNT* and *Nrf2* pathways together with several members of ABC transporters suggesting a key role of these gene products in primary drug resistance of MM cells.

### *PXR*, *CAR*, *LXR* and *FXR* activation may confer drug sensitivity to MMCs

The overexpression of genes coding for the main actors of the DMC system in MMCs of patients with a good outcome could appear somewhat surprising given the well established role of DMEs and transporters to promote the metabolism, elimination and detoxification of chemotherapeutic drugs leading to reduced therapeutic effectiveness and treatment failure. However, emerging reports have demonstrated that xenobiotic receptors as well as their transcriptional targets among DMEs and drug carriers could confer, in a context-dependent manner, either drug resistance or drug sensitivity.

### RXRα

*RXR*α (RXRA), − whose gene expression in MMCs is a good prognostic marker - is the permissive combinatory partner for *PXR, CAR, LXR*, and *FXR* heterodimerization and nuclear translocation after ligand binding. Its activation has been previously associated with good prognosis in different cancers and *RXRα* synthetic ligands, the rexinoids, have shown promising antitumor activity in preclinical and clinical studies in different cancer types [20]. In particular, bexarotene, a synthetic rexinoid, approved by FDA was found to induce a 50% overall inhibitory response in patients with refractory or persistent cutaneous T-cell lymphoma and to improve the overall survival [21]. Several studies have demonstrated that RXRA antitumor activity is attributable to enhanced apoptosis, cell cycle regulation and increased sensitivity to several chemotherapeutic drugs through down regulation of Nrf2 antioxidant pathway [22,23].

### PXR

*PXR* pathway is one of the major xenobiotic signalling cascades enriched in MMCs of low DMC score patients with good outcome. *PXR* is a major coordinator of the detoxification process; but its role in tumor cells is somehow controversial since *PXR* functions in a tissue-specific and/or ligand-promoter dependent manner and could play a key role in chemoresistance or chemosensitivity. The role of *PXR* in promoting chemoresistance is well documented in breast, endometrial, prostate, ovarian colon and colorectal [16]. However, others studies have shown that *PXR* could have anticancer activities, independently of its role in the DMC system, in colon, liver and breast cancers [24,25]. As such, in addition to its master role in drug metabolism regulation, *PXR* is thought to modulate cancer cell resistance or sensitivity through the control of several cellular processes including regulation of genes involved in cell proliferation, metastasis, pro-apoptosis, anti-apoptosis and endocrine homeostasis as well as regulation of the oxidative stress response [18,26,27].

### LXR

In addition to *PXR* pathway, *LXR* pathway is also enriched in low DMC score MMCs. *LXR*, activated by naturally occurring oxysterols, small lipophilic molecules and xenochemicals, act as cholesterol sensors to regulate the transcription of gene products that control intracellular cholesterol homeostasis through biosynthesis, catabolism and transport. The role of *LXR* in cancer drugs response has not been investigated. However, recently, it have demonstrated that activation of *LXR* pathway induces inhibition of clonogenic MM growth, MM tumor initiating cells *in vitro* and *in vivo* [28].

### SLC transporters

A possible explanation of the transcriptomic profile with high drug metabolism and detoxification abilities in MMCs of patients with good outcome is likely due to the fact that high xenobiotic receptors expression trigger upregulation of uptake transporters, the SLC members. Indeed, in those low DMC score MMCs, the expression of 19 SLC members was up regulated, suggesting that drugs might enter more efficiently into MMCs and induce cancer cell death. On the contrary, in high DMC score MMCs, the low expression of xenobiotic receptors and associated low expression of these intake transporters could lead to reduced drug internalization and chemotherapy inefficacy. In particular, low DMC score MMCs highly expressed genes coding for SLC7 family, SLC22 or SLC10, which are crucial for drug uptake [14], [7,29]. Moreover, *SLC7A7* is a major influx transporter of melphalan [30,31], a main drug used to treat MM patients of the HM cohort used in this study. Previous work has shown that down-regulation of *SLC7A7* reduced the Melphalan uptake by 58% and toxicity by 3.5-fold in hematopoietic tumor cells [32]. Further, Agnelli et al (2011) also reported that a high expression of *SLC7A7* gene in MMCs positively correlated with a better prognosis [33].

### CYP450

The high expression of 30 out of the 47 known CYP450 coding genes in low DMC score MMCs could be an additional mechanism, which explains the association of active xenobiotic/drug metabolism in MMCs to favourable patients' outcome. Although CYP450 generally leads to increased elimination of drugs and subsequently to therapeutic failure, an increase in CYP450 can also trigger drug biotransformation, generate more cytotoxic intermediate metabolites and have therapeutic benefit. This is particularly true for some drugs used in the treatment of the patients enrolled in this study, including Cyclophosphamide and Thalidomide. Cyclophosphamide, a nitrogen mustard alkylating prodrug, requires metabolic activation and undergoes *CYP2B6/CYP2C9* mediated oxidation to induce DNA damage and cell death in target cancer cells [34,35].

### ABC transporters

Low DMC score MMCs highly expressed genes coding for ABC transporters but these are exclusively members of ABCA and ABCC subfamilies. Conversely, high DMC score MMCs overexpressed genes encoding for members of *ABCB, ABCD, ABCDE* and *ABCF* families. Several studies have reported the role of some ABC transporters in MM drug resistance, mainly that of *ABCB1/MDR1/PgP* [36-38]. However, a clinical trial with a potent and specific inhibitor to ABCB1 (Zosuquidar) did not show any benefit in progression free or overall survival suggesting additional transporters or mechanisms are involved in conferring drug resistance [39]. Our data suggest that several ABC transporters are involved in the primary drug resistance phenotype, which could justify at least in part the limited therapeutic success of monotargeted therapies and imply, in accordance with previous studies, that ABC transporter family could exhibit a high degree of functional redundancy [40,41]. Moreover, our data strongly suggest that in cancer drug resistant or sensitive phenotype, more concern should be given to the ratio of efflux (ABC transporters) versus influx transporters (SLC transporters) rather than considering efflux proteins solely.

Although MMCs of patients with bad outcome display a global down regulation of DMC genes expression, they overexpressed genes coding for *ARNT* or *Nrf2* pathways.

### *ARN*T

*ARNT* also designated as hypoxia-inducible factor *HIF-1β,* is a major transcription factor up regulated in high DMC score MMCs. *ARNT* serves as binding partner for several bHLH members and plays a key role in two distinct cellular signalling pathways – the *AhR* and HIF pathways - activated in response to environment stimulations and also largely involved in cancer cell biology. The HIF pathway mediates cellular adaptive responses to reduced oxygen supply. Under sufficient oxygen supply (normoxia), *HIF1α* is degraded after ubiquitination; under hypoxia, *HIF1α* accumulates, heterodimerizes with ARNT and translocates to the nucleus. The *HIF1α/ARNT* complex mediates the transcription of numerous target genes mediating adaptive response to low oxygen tension. HIF signalling has been shown to contribute to tumor progression by promoting invasion/metastasis, metabolic alterations and the induction of angiogenesis in numerous cancer types. *HIF1*α expression has been reported in about 35% of CD138^+^ cells isolated from MM patient's samples [42]. In preclinical studies, the inhibition of *HIF1α* has been shown to enhance the sensitivity to melphalan and early down-regulation of *HIF1*α expression has been reported in MM cell lines sensitive to bortezomib and lenalidomide [42-44].

*AhR*, the second heterodimerization partner for ARNT, is a chemosensor responsive signalling cascade to a set of environmentally common immunosuppressive chemicals. After ligand binding, *AhR* undergoes a conformational change, forms a heterodimer with *ARNT* and activates the transcription of a wide range of phase I/II DMEs and drug transporters. For several decades, *AhR/ARNT* pathway has been studied largely because of its critical role in immunosuppression and as major regulator of xenobiotic-induced carcinogenesis [10]. Early studies have demonstrated a major role for the *AhR* in malignant B cell transformation including B lymphomas, leukaemia and multiple myeloma among other cancers [45]. More recently, increasing epidemiological and experimental data provided substantial support that *AhR* presumably activated by endogenous ligand(s) plays an important role in tumor promotion and progression [46]. As such, *AhR* was found to be over-expressed and constitutively active in a variety of cancers and its nuclear expression is frequently associated with bad prognosis and advanced histological grade [47,48]. In particular, many studies demonstrated elevated *AhR* levels and constitutive activity in a variety of cancer cell lines and B lineage malignancies including lymphomas, myelomas and T cell leukemia [45]. Microarray analysis of 1,036 human cancer cell lines revealed a significant role of *AhR* in myelomas and other B lineage cancer subtypes among other cancers [49]. In cancer cells, AhR is though to promote cell proliferation, cell motility and migration and oxidative stress through cross talk with *ER, NFkB, p53* and *Nrf2* pathways [50,51].

Using acute myeloid leukaemia cells, recent data pointed out increased antioxidants enzymes and *Nrf2* transcripts and elevated intracellular glutathione concentration after *ARNT* up regulation [52]. Further, it have been demonstrated that the knockdown of *ARNT* in cancer cells reduced proliferation rate and the transformation ability and enhanced cisplatin-induced apoptosis [53].

### *Nrf*2

The second major pathway found up regulated in the high DMC score group with the worst prognosis is *Nrf2* signalling cascade. *Nrf2* is at the crossroad between drug metabolism and oxidative stress responses. This was further confirmed by data from Ingenuity Pathways Analysis and GSEA analysis underlining oxidative stress as major component of high DMC score group signature.

In unstressed cells, *Nrf2* is bound to *Keap1*, ubiquitinated and degraded by proteasome. Upon electrophilic stress, *Nrf2* is released from Keap1, translocates into the nucleus and activates the transcription of genes coding for redox balancing proteins (heme-oxygenase1), phase II detoxifying genes and drug transporters [54]. Growing evidences suggest that a *Nrf2* constitutive up-regulation is associated with cancer development, progression and resistance to chemotherapy [55-58]. Constitutive activation of *Nrf2* is a major advantage for cancer cell for detoxification of ROS associated with cell cycle and growth. The oncogenes *K-Ras, B-Raf,* and *Myc* can stimulate *Nrf2* gene transcription in cancer cells, leading to a reduction in the intracellular ROS level and the promotion of oncogenesis. Interestingly, elevated activity of *Nrf2* in cancer cells has been shown to decrease their sensitivity to common chemotherapeutic agents. This is particularly true for the proteasome inhibitor Bortezomib, a major line of MM treatment. *In vitro* and in clinical studies showed high expression of *Nrf2* associated with poor responsiveness to Bortezomib treatment [59,60]. In accordance with other reports, our data underlined a key role of oxidative stress in MM prognosis with the involvement with several signalling pathways related to redox homeostasis [61-65].

The current study shows that the genes coding for xenobiotic receptors and their downstream DMC target genes are overexpressed in MMCs of patients with good outcome and only those coding for *ARNT or Nrf2* pathways in MMCs of patients with poor outcome. Above all, these data suggest that selectively targeting upstream regulators of the major *PXR, FXR, LXR* and *Nrf2* pathways using a subset of synthetic antagonists or agonists for those xenobiotic receptors could provide a promising strategy to improve the efficacy of treatment in MM. A fine understanding of the DMC system in MM biology will help improving the use of drugs currently used in MM.

## METHODS

### Patient samples and gene expression data

206 patients with newly-diagnosed MM were enrolled in the current study after written informed consent at the University hospitals of Heidelberg (Germany) or Montpellier (France) (HM cohort). These patients underwent frontline induction treatment with Dexamethasone and various drugs, high-dose chemotherapy with 200 mg/m2 Melphalan and autologous stem cell transplantation according or in analogy to the GMMG-HD3-trial [66]. At relapse, various treatments regimens were applied including Bortezomib and immunomodulatory drugs (Thalidomide, Lenalidomide, Pomalidomide).

Bone marrow was harvested at diagnosis, MMCs were purified, gene expression profiling (GEP) assayed using Affymetrix U133 2.0 plus microarrays, and data normalized using the MAS5 Affymetrix algorithm with a scaling factor of 500 as described previously [67]. The .CEL and MAS5 files are deposited in the Array Express public database (http://www.ebi.ac.uk/arrayexpress/) under accession number E-MTAB-362.

Publicly available MAS5 normalized GEP data (GEO, http://www.ncbi.nlm.nih.gov/geo/, accession number GSE2658) from purified MMCs of a cohort of 345 patients were also used. These patients were treated with total therapy 2 protocol (UAMS-TT2 cohort) at the University of Arkansas for Medical Sciences (UAMS, Little Rock, USA) [68].

### Identification of prognostic Drug Metabolism and Clearance genes (DMC genes) and DMC score building

Based on the review of literature and databases [69], a consensus list of 350 human genes coding for xenobiotic receptors and co-regulators (29 genes), phase I DMEs (107 genes), phase II DMEs (90 genes) and uptake and efflux transporters (124 genes) was selected. The corresponding Affymetrix probe sets and gene ontology description are listed in the Supplemental Table S1. When several probe sets were available for a same gene, the probe set with the highest variance was chosen.

Genes whose expression in MMCs could predict for patients' Event Free Survival (EFS) were identified using a univariate Cox Model. A prognostic Drug Metabolism and Clearance score (termed DMC score) was built by computing the mean of the standardized Affymetrix signals of the prognostic genes weighted by their Cox Beta Coefficient. Then patients were ranked according to increased DMC score and split into 3 groups according to their expression of the prognostic DMC genes in MMCs using the k-means function.

### Data Analysis

The analyses were done with R (http://www.r-project.org/) and Bioconductor (http://www.bioconductor.org/) softwares. Survival curves were plotted using the Kaplan-Meier method. Gene expression data were visualized using Cluster (v2.11) and Tree View (v1.6, Eisen laboratory, Berkeley, USA). Ingenuity Pathway Analysis (IPA) software was carried out using a false discovery rate (FDR < 0.05) with at least 5 genes for one pathway. Gene set enrichment analysis was performed using the GSEA Software (http://www.broadinstitute.org/gsea/index.jsp, Broad Institute, Cambridge, USA), and the collections for canonical pathways (c2.cp.kegg) or transcription factor targets (C3.tft).

## SUPPLEMENTARY TABLE



## References

[1] Morgan GJ, Walker BA, Davies FE (2012). The genetic architecture ofmultiple myeloma. Nature Publishing Group. Nature Publishing Group.

[2] Raab MS, Podar K, Breitkreutz I, Richardson PG, Anderson KC (2009). Multiple myeloma. The Lancet.

[3] Rajkumar SV, Gahrton G, Bergsagel PL (2011). Approach to the treatment of multiple myeloma: a clash of philosophies. Blood.

[4] Chen Y, Tang Y, Guo C, Wang J, Boral D, Nie D (2012). Biochemical Pharmacology. Biochem. Pharmacol.

[5] Akhdar H, Legendre C, Aninat C, More F, Paxton James Anticancer Drug Metabolism: Chemotherapy Resistance and New Therapeutic Approaches, Topics on Drug Metabolism.

[6] Ma Q (2008). Xenobiotic-Activated Receptors: From Transcription to Drug Metabolism to Disease. Chem. Res. Toxicol.

[7] Döring B, Petzinger E (2014). Phase 0 and phase III transport in various organs: Combined concept of phases in xenobiotic transport and metabolism. Drug Metabolism Reviews.

[8] Omiecinski CJ, Vanden Heuvel JP, Perdew GH, Peters JM (2011). Xenobiotic Metabolism, Disposition, and Regulation by Receptors: From Biochemical Phenomenon to Predictors of Major Toxicities. Toxicological Sciences.

[9] Conney AH (2003). I NDUCTION OFD RUG-M ETABOLIZINGE NZYMES: A Path to the Discovery of Multiple Cytochromes P450*. Annu. Rev. Pharmacol. Toxicol.

[10] Zanger UM, Schwab M (2013). Pharmacology & Therapeutics. Pharmacology and Therapeutics.

[11] Gamage N (2005). Human Sulfotransferases and Their Role in Chemical Metabolism. Toxicological Sciences.

[12] Wells PG, Mackenzie PI, Chowdhury JR, Guillemette C, Gregory PA, Ishii Y, Hansen AJ, Kessler FK, Kim PM, Chowdhury NR, Ritter JK (2004). Drug Metab Dispos.

[13] Scotto KW (2003). Transcriptional regulation of ABC drug transporters. Oncogene.

[14] Zamek-Gliszczynski MJ, Hoffmaster KA, Tweedie DJ, Giacomini KM, Hillgren KM (2012). Highlights from the International Transporter Consortium Second Workshop. Clin Pharmacol Ther.

[15] Yu M, Ocana A, Tannock IF (2012). Reversal of ATP-binding cassette drug transporter activity to modulate chemoresistance: why has it failed to provide clinical benefit?. Cancer Metastasis Rev.

[16] Pondugula SR, Mani S (2013). Pregnane xenobiotic receptor in cancer pathogenesis and therapeutic response. Cancer Letters.

[17] Walsh JS, Miwa GT (2011). Bioactivation of Drugs: Risk and Drug Design. Annu. Rev. Pharmacol. Toxicol.

[18] Tang J (2013). Expression of the PXR gene in various types of cancer and drug resistance (Review). Oncol Lett.

[19] Gold LS, De Roos AJ, Brown EE, Lan Q, Milliken K, Davis S (2009). Associations of common variants in genes involved in metabolism and response to exogenous chemicals with risk of multiple myeloma. Cancer Epidemiology.

[20] Tanaka T, De Luca LM (2009). Therapeutic Potential of “Rexinoids” in Cancer Prevention and Treatment. Cancer Research.

[21] Gniadecki R, Assaf C, Bagot M, Dummer R, Duvic M, Knobler R (2007). The optimal use of bexarotene in cutaneous T-cell lymphoma. Br. J. Dermatol.

[22] Evans RM, Mangelsdorf DJ (2014). Nuclear Receptors, RXR, and the Big Bang. Cell.

[23] Chorley BN, Campbell MR, Wang X, Karaca M, Sambandan D, Bangura F (2012). Identification of novel NRF2-regulated genes by ChIP-Seq: influence on retinoid X receptor alpha. Nucleic Acids Res.

[24] Ouyang N, Ke S, Eagleton N, Xie Y, Chen G, Laffins B (2010). Pregnane X receptor suppresses proliferation and tumourigenicity of colon cancer cells. British Journal of Cancer.

[25] Verma S, Tabb MM, Blumberg B (2009). Activation of the steroid and xenobiotic receptor, SXR, induces apoptosis in breast cancer cells. BMC Cancer.

[26] Zhuo W, Hu L, Lv J, Wang H, Zhou H, Fan L (2014). Role of pregnane X receptor in chemotherapeutic treatment. Cancer Chemother Pharmacol.

[27] Robbins D, Chen T (2014). Tissue-specific regulation of pregnane X receptor in cancer development and therapy. Cell & Bioscience. Cell & Bioscience.

[28] Agarwal JR, Wang Q, Tanno T, Rasheed Z, Merchant A, Ghosh N (2014). Activation of Liver X Receptors Inhibits Hedgehog Signaling, Clonogenic Growth, and Self-Renewal in Multiple Myeloma. Molecular Cancer Therapeutics.

[29] Petzinger E, Geyer J (2006). Drug transporters in pharmacokinetics. Naunyn Schmiedebergs Arch. Pharmacol.

[30] Lin J, Raoof DA, Thomas DG, Greenson JK, Giordano TJ, Robinson GS (2004). L-type amino acid transporter-1 overexpression and melphalan sensitivity in Barrett's adenocarcinoma. Neoplasia.

[31] Kühne A, Kaiser R, Schirmer M, Heider U, Muhlke S, Niere W (2007). Genetic polymorphisms in the amino acid transporters LAT1 and LAT2 in relation to the pharmacokinetics and side effects of melphalan. Pharmacogenet Genomics.

[32] Kühne A, Tzvetkov MV, Hagos Y, Lage H, Burckhardt G, Brockmöller J (2009). Influx and efflux transport as determinants of melphalan cytotoxicity: Resistance to melphalan in MDR1 overexpressing tumor cell lines. Biochem. Pharmacol.

[33] Agnelli L, Forcato M, Ferrari F, Tuana G, Todoerti K, Walker BA (2011). The reconstruction of transcriptional networks reveals critical genes with implications for clinical outcome of multiple myeloma. Clin. Cancer Res.

[34] Huang Z, Roy P, Waxman DJ (2000). Role of human liver microsomal CYP3A4 and CYP2B6 in catalyzing N-dechloroethylation of cyclophosphamide and ifosfamide. Biochem. Pharmacol.

[35] Wang D, Li L, Yang H, Ferguson SS, Baer MR, Gartenhaus RB (2013). The constitutive androstane receptor is a novel therapeutic target facilitating cyclophosphamide-based treatment of hematopoietic malignancies. Blood.

[36] Tsubaki M, Satou T, Itoh T, Imano M, Komai M, Nishinobo M (2012). Overexpression of MDR1 and survivin, and decreased Bim expression mediate multidrug-resistance in multiple myeloma cells. Leukemia Research.

[37] Tsubaki M, Komai M, Itoh T, Imano M, Sakamoto K, Shimaoka H (2014). By inhibiting Src, verapamil and dasatinib overcome multidrug resistance via increased expression of Bim and decreased expressions of MDR1 and survivin in human multidrug-resistant myeloma cells. Leukemia Research.

[38] Martino A, Sainz J, Reis RM, Moreno V, Buda G, Lesueur F (2013). Polymorphisms in regulators of xenobiotic transport and metabolism genes PXR and CAR do not affect multiple myeloma risk: a case–control study in the context of the IMMEnSE consortium. Journal of Human Genetics.

[39] Friedenberg WR, Rue M, Blood EA, Dalton WS, Shustik C, Larson RA (2006). Phase III study of PSC-833 (valspodar) in combination with vincristine, doxorubicin, and dexamethasone (valspodar/VAD) versus VAD alone in patients with recurring or refractory multiple myeloma (E1A95): a trial of the Eastern Cooperative Oncology Group. Cancer.

[40] Januchowski R, Zawierucha P, Andrzejewska M, Ruciński M, Zabel M (2013). Microarray-based detection and expression analysis of ABC and SLC transporters in drug-resistant ovarian cancer cell lines. Biomed. Pharmacother.

[41] Szakács G, Annereau J-P, Lababidi S, Shankavaram U, Arciello A, Bussey KJ (2004). Predicting drug sensitivity and resistance: profiling ABC transporter genes in cancer cells. Cancer Cell.

[42] Colla S, Storti P, Donofrio G, Todoerti K, Bolzoni M, Lazzaretti M (2010). Low bone marrow oxygen tension and hypoxia-inducible factor-1α overexpression characterize patients with multiple myeloma: role on the transcriptional and proangiogenic profiles of CD138(+) cells. Leukemia.

[43] Storti P, Bolzoni M, Donofrio G, Airoldi I, Guasco D, Toscani D (2013). Hypoxia-inducible factor (HIF)-1α suppression in myeloma cells blocks tumoral growth *in vivo* inhibiting angiogenesis and bone destruction. Leukemia.

[44] Hu Y, Kirito K, Yoshida K, Mitsumori T, Nakajima K, Nozaki Y (2009). Inhibition of hypoxia-inducible factor-1 function enhances the sensitivity of multiple myeloma cells to melphalan. Molecular Cancer Therapeutics.

[45] Sherr DH, Monti S (2013). The role of the aryl hydrocarbon receptor in normal and malignant B cell development. Semin Immunopathol.

[46] Feng S, Cao Z, Wang X, Elsevier B.V (2013). Biochimica et Biophysica Acta. BBA - Reviews on Cancer.

[47] Casado FL, Singh KP, Gasiewicz TA (2010). Blood Cells, Molecules, and Diseases. Blood Cells, Molecules, and Diseases.

[48] Safe S, Lee SO, Jin UH (2013). Role of the Aryl Hydrocarbon Receptor in Carcinogenesis and Potential as a Drug Target. Toxicological Sciences.

[49] Barretina J, Caponigro G, Stransky N, Venkatesan K, Margolin AA, Kim S (2012). The Cancer Cell Line Encyclopedia enables predictive modelling of anticancer drug sensitivity. Nature.

[50] Haarmann-Stemmann T, Abel J, Fritsche E, Krutmann J (2012). The AhR-Nrf2 pathway in keratinocytes: on the road to chemoprevention? J. Invest. Dermatol.

[51] Shin S, Wakabayashi N, Misra V, Biswal S, Lee GH, Agoston ES (2007). NRF2 modulates aryl hydrocarbon receptor signaling: influence on adipogenesis. Mol. Cell. Biol.

[52] Gu C, Gonzalez J, Zhang T, Kamel-Reid S, Wells RA (2013). Leukemia Research. Leukemia Research.

[53] Shieh J-M, Shen C-J, Chang W-C, Cheng H-C, Chan Y-Y, Huang W-C, Chang Y-J (2014). An Increase in Reactive Oxygen Species by Deregulation of ARNT Enhances Chemotherapeutic Drug-Induced Cancer Cell Death. PLoS ONE.

[54] Sporn MB, Liby KT (2012). NRF2 and cancer: the good, the bad and the importance of context. Nat Rev Cancer.

[55] Konstantinopoulos PA, Spentzos D, Fountzilas E, Francoeur N, Sanisetty S, Grammatikos AP (2011). Keap1 mutations and Nrf2 pathway activation in epithelial ovarian cancer. Cancer Research.

[56] Hayes JD, McMahon M (2006). The double-edged sword of Nrf2: subversion of redox homeostasis during the evolution of cancer. Mol. Cell.

[57] Hayes JD, Dinkova-Kostova AT, McMahon M (2009). Cross-talk between transcription factors AhR and Nrf2: lessons for cancer chemoprevention from dioxin. Toxicological Sciences.

[58] Wang X-J, Sun Z, Villeneuve NF, Zhang S, Zhao F, Li Y (2008). Nrf2 enhances resistance of cancer cells to chemotherapeutic drugs, the dark side of Nrf2. Carcinogenesis.

[59] Rushworth SA, Bowles KM, MacEwan DJ (2011). High Basal Nuclear Levels of Nrf2 in Acute Myeloid Leukemia Reduces Sensitivity to Proteasome Inhibitors. Cancer Research.

[60] Weniger MA, Rizzatti EG, Perez-Galan P, Liu D, Wang Q, Munson PJ (2011). Treatment-Induced Oxidative Stress and Cellular Antioxidant Capacity Determine Response to Bortezomib in Mantle Cell Lymphoma. Clinical Cancer Research.

[61] Sharma A, Tripathi M, Satyam A, Kumar L (2009). Study of antioxidant levels in patients with multiple myeloma. Leuk Lymphoma.

[62] Stellrecht CM, Gandhi V (2009). Myeloma antioxidant status: the good, the bad and the reactive. Leuk Lymphoma.

[63] Gangemi S, Allegra A, Alonci A, Cristani M, Russo S, Speciale A (2012). Increase of novel biomarkers for oxidative stress in patients with plasma cell disorders and in multiple myeloma patients with bone lesions. Inflamm. Res.

[64] Goel A, Spitz DR, Weiner GJ (2012). Manipulation of cellular redox parameters for improving therapeutic responses in B-cell lymphoma and multiple myeloma. J. Cell. Biochem.

[65] Nerini-Molteni S, Ferrarini M, Cozza S, Caligaris-Cappio F, Sitia R (2008). Redox homeostasis modulates the sensitivity of myeloma cells to bortezomib. Br J Haematol.

[66] Goldschmidt H, Sonneveld P, Cremer FW, van der Holt B, Westveer P, Breitkreutz I (2003). Joint HOVON-50/GMMG-HD3 randomized trial on the effect of thalidomide as part of a high-dose therapy regimen and as maintenance treatment for newly diagnosed myeloma patients. Ann Hematol.

[67] De Vos J, Thykjaer T, Tarte K, Ensslen M, Raynaud P, Requirand G, Pellet F, Pantesco V, Reme T, Jourdan M, Rossi JF, Klein B (2002). Comparison of gene expression profiling between malignant and normal plasma cells with oligonucleotide arrays. Oncogene.

[68] Barlogie B (2006). Total therapy 2 without thalidomide in comparison with total therapy 1: role of intensified induction and posttransplantation consolidation therapies. Blood.

[69] Yang L, Price ET, Chang C-W, Li Y, Huang Y, Guo L-W, Miao X-P (2013). Gene Expression Variability in Human Hepatic Drug Metabolizing Enzymes and Transporters. PLoS ONE.

